# The impact of the cost-of-living crisis on population health in the UK: rapid evidence review

**DOI:** 10.1186/s12889-024-17940-0

**Published:** 2024-02-22

**Authors:** Jade Meadows, Miranda Montano, Abdelrahman J. K. Alfar, Ömer Yetkin Başkan, Caroline De Brún, Jennifer Hill, Rachael McClatchey, Nevila Kallfa, Gwen Sascha Fernandes

**Affiliations:** 1 South West Critical Thinking Unit, Health Care Public Health Directorate, NHS England, Bristol, United Kingdom; 2 Public Health Intelligence Team, Devon County Council, Exeter, England; 3 Global Business School for Health (GBSH), University College London (UCL), London, United Kingdom; 4 Knowledge and Library Services, UK Health Security Agency, London, United Kingdom; 5 Office for Health Improvement and Disparities, DHSC, Bristol, United Kingdom; 6 Faculty of Health and Applied Sciences, University of the West of England, Bristol, England; 7 Faculty of Medicine & Health Sciences, University of Nottingham, Nottingham, England

**Keywords:** cost of living, population health outcomes, physical health, mental health

## Abstract

**Background:**

In the UK, unique and unforeseen factors, including COVID-19, Brexit, and Ukraine-Russia war, have resulted in an unprecedented cost of living crisis, creating a second health emergency. We present, one of the first rapid reviews with the aim of examining the impact of this current crisis, at a population level. We reviewed published literature, as well as grey literature, examining a broad range of physical and mental impacts on health in the short, mid, and long term, identifying those most at risk, impacts on system partners, including emergency services and the third sector, as well as mitigation strategies.

**Methods:**

We conducted a rapid review by searching PubMed, Embase, MEDLINE, and HMIC (2020 to 2023). We searched for grey literature on Google and hand-searched the reports of relevant public health organisations. We included interventional and observational studies that reported outcomes of interventions aimed at mitigating against the impacts of cost of living at a population level.

**Results:**

We found that the strongest evidence was for the impact of cold and mouldy homes on respiratory-related infections and respiratory conditions. Those at an increased risk were young children (0–4 years), the elderly (aged 75 and over), as well as those already vulnerable, including those with long-term multimorbidity. Further short-term impacts include an increased risk of physical pain including musculoskeletal and chest pain, and increased risk of enteric infections and malnutrition. In the mid-term, we could see increases in hypertension, transient ischaemic attacks, and myocardial infarctions, and respiratory illnesses. In the long term we could see an increase in mortality and morbidity rates from respiratory and cardiovascular disease, as well as increase rates of suicide and self-harm and infectious disease outcomes. Changes in behaviour are likely particularly around changes in food buying patterns and the ability to heat a home. System partners are also impacted, with voluntary sectors seeing fewer volunteers, an increase in petty crime and theft, alternative heating appliances causing fires, and an increase in burns and burn-related admissions. To mitigate against these impacts, support should be provided, to the most vulnerable, to help increase disposable income, reduce energy bills, and encourage home improvements linked with energy efficiency. Stronger links to bridge voluntary, community, charity and faith groups are needed to help provide additional aid and support.

**Conclusion:**

Although the CoL crisis affects the entire population, the impacts are exacerbated in those that are most vulnerable, particularly young children, single parents, multigenerational families. More can be done at a community and societal level to support the most vulnerable, and those living with long-term multimorbidity. This review consolidates the current evidence on the impacts of the cost of living crisis and may enable decision makers to target limited resources more effectively.

**Supplementary Information:**

The online version contains supplementary material available at 10.1186/s12889-024-17940-0.

## Introduction

**Fig. 1:**
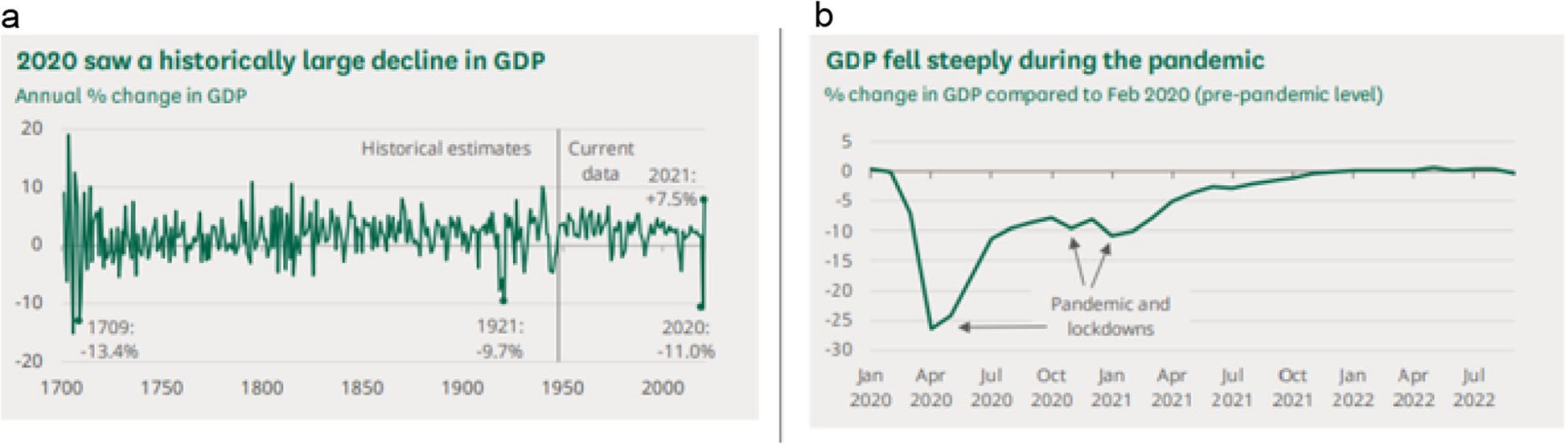
Panel **a**: Annual % change in UK GDP since records began; Panel **b**: % change in GDP compared to Feb 2020 (pre-pandemic levels) [1].

In the UK, the COVID -19 pandemic and subsequent unforeseen geopolitical factors (e.g., Brexit & Ukraine-Russia War) resulted in a severe economic downturn with gross domestic product (GDP) decreasing by 11.0% in 2020, the sharpest drop since records began and unprecedented in modern times (Fig. 1, panel a). Since March 2020, whilst GDP has increased*,* it has remained below 2020 pre-pandemic levels through to July 2022, accompanied by rising inflation rates due to endogenous and exogenous shocks (Fig. 1, panel b) [1]. Internal shocks include supply chain challenges or labour shortages which affected the supply side, and pandemic-associated changes in consumer purchasing patterns which affected the demand side. These internal shocks have resulted in imbalances between the supply and demand of different markets including the goods and services market, the labour market, and the money market. Exogenous shocks occur due to non-economic interventions such as war (e.g., the Ukraine-Russia war) and the ongoing global pandemics (e.g., COVID-19). This combination of economic shocks is unprecedented, therefore the resulting impacts on inflation and the cost of living (CoL) are unique, both in terms of provenance and consequences for population health and wellbeing. This is further exacerbated by COVID-19 consequences and recovery from the pandemic which has been estimated to take 10–15 years [2]. The cost of living crisis is often regarded as the ‘second health emergency’ after the COVID-19 pandemic [3].

Recent evidence from the World Health Organisation (WHO) shows that economic shocks and austerity exacerbate poverty, vulnerability, marginalisation, as well as socioeconomic and health inequalities [4–7], with serious implications for health [2, 8],. In the UK, rising inflation has contributed to the rising CoL, which has made the population poorer and driven 1 in 5 into relative poverty [9].

**Fig. 2:**
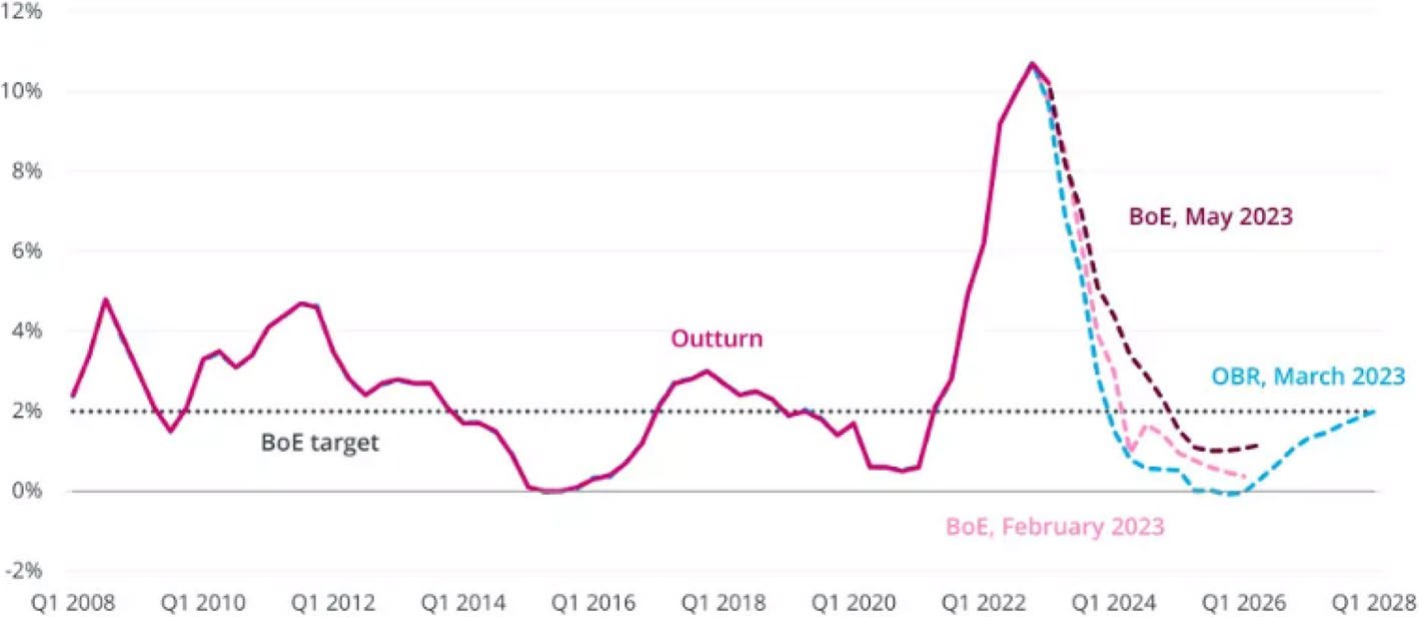
CPI inflation Q1 2008 to Q1 2028, including successive Bank of England and Office for Budget Responsibility forecasts. Source: Institute for Government

The UK Office for National Statistics (ONS) checks the prices of a whole range of items in a standard ‘basket’ of goods and services and the price of that basket determines the overall price level, otherwise known as the Consumer Prices Index (CPI). Inflation is a term used to describe rising prices and how quickly prices go up is called the rate of inflation. To calculate the inflation rate, the cost of the basket or level of CPI is compared with the previous year, and this change in price level over the year is the rate of inflation [10, 11]. The inflation rate in the UK (February 2023) was 10.5% against a usual target of 2% [10]. The inflation rate is projected to worsen through 2023 and subsequently decrease by Q1 of 2024 as depicted in Fig. 2 [10], a trend confirmed in recent months with inflation now at 4.2% (Jan 2024).

According to the official economic projections, the expected UK economic recovery is slower than the G7 countries, a full recovery and return to pre-pandemic peak is expected in the end of 2024. The average annual growth rate is less than 1% (per year), from the start of the pandemic till 2028. Prior to this period, the growth rate was 2.75%. In addition to this, the real disposable income per person is expected to fall by 5.7 cumulative percent by the end of the next March 2024 [12].

Meanwhile the consumer’s wages and benefit payments are not in tandem with rising living costs, and in particular, the cost of housing, food, energy, and fuel [13]. For example, evidence suggests that 14.4% of households in the UK (approximately 3.53 million) will be living in fuel poverty by January 2023 [14]. Those at risk include large families, lone parents, and pensioner couples [15], with the elderly and children being most vulnerable to an increased risk of physical illness such as respiratory infections [16]. In addition, studies have found that economic hardships increase the prevalence of mental illness, including feelings of anxiety and depression [17]. The modest economic growth, the decline in real disposable income, and the slow recovery expectations will put more pressure on hospital admission from respiratory and mental illnesses.

To better understand the current cost of living crisis, the associated triggers, drivers, consequences and potential solutions, a rapid evidence review was undertaken between November 2022 and March 2023. This was a cross-organisation, multidisciplinary endeavour that brought together academics, researchers, clinicians, public health practitioners, and policymakers to develop a review that would be informative, useful and of value to the wider health and care system and their stakeholders.

## Aims and objectives

The review aimed to provide a first narrative review of evidence relevant to the current CoL crisis and impact on our population health, wellbeing and related services.

The objectives of this evidence review were:

1. To explain the provenance of the current CoL crisis from a UK perspective including the causes, triggers, drivers, and consequences.
2. To map the short (1 – 2 months), mid- (3 – 6 months) and long- term (6 months plus) implications of the current CoL crisis on population health and wellbeing. These would be themed by physical health, mental health, wellbeing, education, environment, workforce, and wider health and care system pressures. It would also involve the identification of those most vulnerable to the cost-of-living crisis.
3. To describe how people change their behaviour or cope/respond to the current CoL crisis, how it impacts on their self-care (e.g., attending regular health checks or screening visits) and the longer-term implications of this behaviour change.
4. To describe some of the mitigation strategies or interventions that have been deployed to counter the negative impacts of the current CoL/economic crisis and their effects in the UK and key European counterparts, where available.
5. To describe the breadth of impacts on health and care system partners of the current CoL crisis.

## Methodology

### Search strategy

The search strategy was based on the objectives of this review and cross-referenced with the search strategy adopted by UKHSA’s Library Services on CoL and poverty evidence reviews, conducted since November 2022. Studies published from 2020, which was the start of the coronavirus pandemic and unique set of circumstances culminating in the cost of living crisis, and up to and including the 2023 were reviewed.

Key themes were discussed with experts, such as respiratory consultants, to finalise the search strategy to ensure the search included key phrases.

The definition of the CoL crisis has been taken from the WHO which describes CoL as the decrease in real disposable income that people have been experiencing since late 2021. The key causes are high inflation overriding income and benefit increases, and, have been worsened by the COVID-19 pandemic, the war in Ukraine, disruption to global supply chains and the food and energy crises [2].

The keywords included: cost of living, population impacts, living cost, fuel poverty, poverty, mental health, physical health, wellbeing, education, work environment, ability to work, and ability to care. Full details of search strategy are included in Appendix 1.

## Selection methodology

### Evidence identification, screening and extraction

The selection criteria were structured around PICOS structure [18, 19]:

#### Population

All age groups impacted by the CoL crisis in the UK.

#### Intervention

Any strategies or interventions that have been used to mitigate the impacts of a CoL crisis to be presented by theme/topic, individual level e.g., reducing energy bills at home or changing food preparation patterns; community level e.g., provision of warm drinks at community centres for the elderly, economic/societal level e.g., energy payments for all households, etc. Could also present these at local/sub-regional, national levels.

#### Comparator

All age groups that are used to compare with and against those most impacted by CoL (those most protected or least vulnerable to a financial crisis).

#### Outcomes

The primary outcomes includes a comprehensive tally of physical health (e.g., admissions, morbidity, mortality), Mental Health (e.g., anxiety, depression, injuries, suicide and self-harm), Wellbeing (e.g., social and economic insecurity, working additional jobs, reduced leisure time), educational attainment/school absenteeism, Environmental (e.g., pollution, housing conditions, infrastructure disruptions), workforce (inability to work due to sickness inability to care for family members) and Service pressures or impacts (e.g., lack of staff, lack of community based care or service provision).

#### Study design

Due to the specific nature of this CoL crisis and time restrictions, we reviewed all review study designs.

### Inclusion criteria

UK-based studies only, due to the unique set of circumstances political policies and populations pertaining to the UK; review studies from the 1st of January 2020 up to include the 24th of February 2023. Including grey literature, such as reports from think tanks and charities, such as The Kings Fund [3] and the Joseph Rowntree Foundation [9, 20].

### Exclusion criteria

Non-UK based studies, specific study formats including letters to Editors.

### Article review

**Fig. 3:**
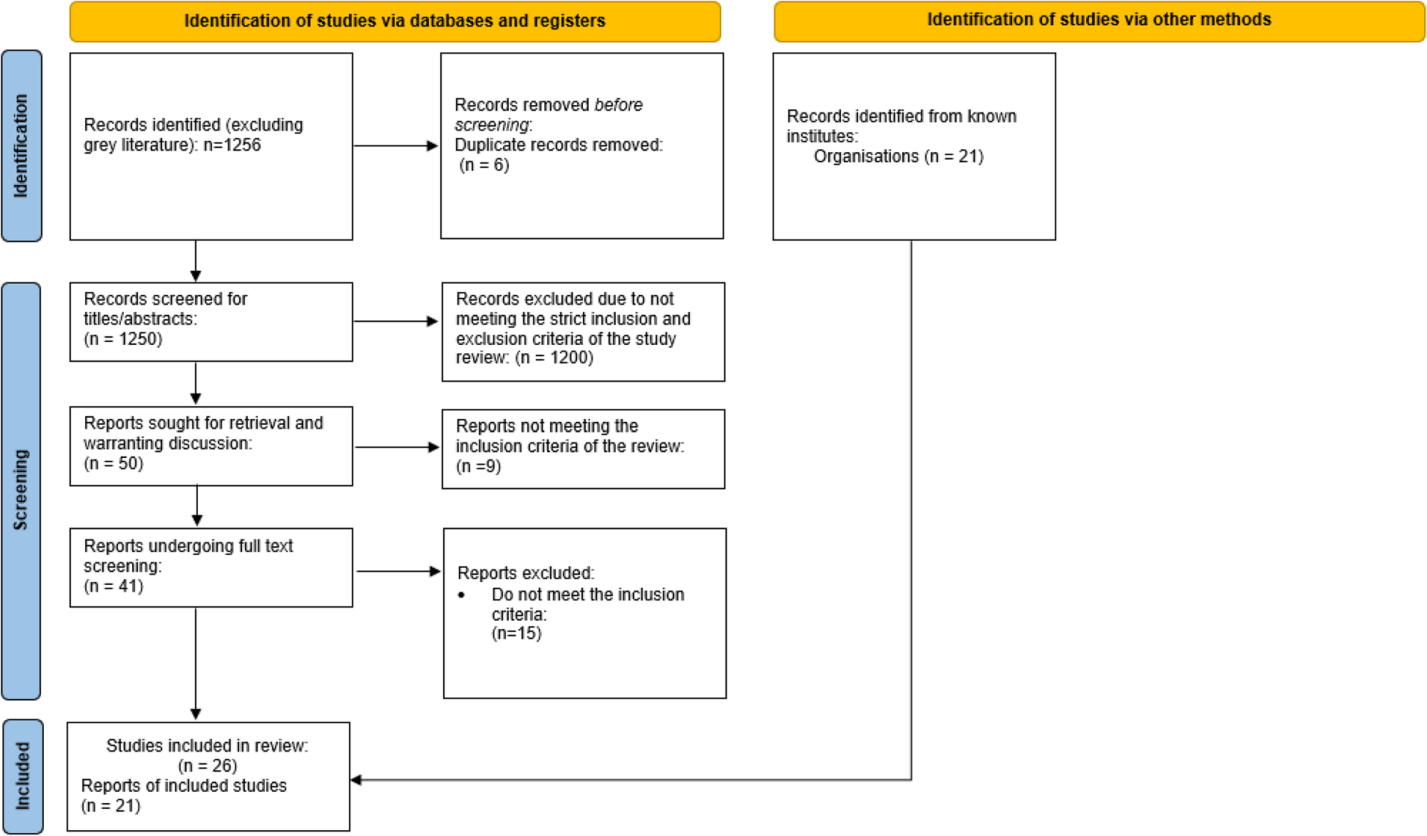
PRISMA flowchart showing inclusion of studies into the study review process through Rayyan including identification, screening and final, included articles

From the search, excluding grey literature, 1,256 records were identified. The team extracted these titles/abstracts into Rayyan, an online software programme used for systematic literature reviews. The study team were then given individual access, with the ‘blind’ feature activated, allowing for independent assessments to be made of each article and corresponding eligibility for inclusion into this evidence review. The initial abstract review was undertaken by two members of the study team, with 50% of articles assessed by at least two members of the team – any discrepancies were discussed and a mutual decision made. Following abstract review, the full text screen was divided into topic areas where two team members reviewed each paper for suitability. As previously, any discrepancies were discussed and a mutual decision made. Each extracted article, regardless of relevance or quality, underwent initial screening to determine relevance to the review topic. Figure 3 details the process for inclusion of studies.

### Quality assessment methodology

Forty-one studies were included in the full text review. Suitable studies (with interventions) were appraised for quality by a primary reviewer and, to ensure robustness, 50% of these were appraised by a second reviewer. Study quality (cross-sectional, case–control, cohort or qualitative) was assessed using the Newcastle Ottawa Scale (NOS) adapted to consider key areas: selection (representativeness of the sample, sample size, non-responders and exposure details); comparability (is confounding considered) and outcome (blinding, recording and statistical test used). Whilst there are a myriad range of assessment tools available, the NOS has been endorsed by the Cochrane Collaboration to assess quality of research studies [18].

Three studies were eligible for scoring, the results are detailed in Table 1. Eligibility for scoring was based on whether an intervention was evaluated as part of the study – those that described an intervention were considered for scoring.

**Table 1:** Summary of papers identified from literature review

Title	Year	Authors	Study design	CoL Theme and focus
Cost of living crisis: a UK crisis with global implications—A call to action for paediatricians	2022	Singh G and Uthayakumar-Cumarasamy A	Review and commentary	Short, mid and long term health implications- children
Obesity and the cost of living crisis	2022	Robinson E	Review and commentary	Short, mid and long term health implications – population health
Cost of living crisis is a threat to good health	2023	Yeung CA and Dickson K	Review and commentary	Short, mid and long term health implications- children
The cost-of-living crisis, poverty, and child maltreatment	2023	Skinner G and Bywaters P and Kennedy E	Review and commentary	Short, mid and long term health implications- children
New evidence on the impact of the Great Recession on health-compromising behaviours	2021	Hall J et.al	Longitudinal study	Behavioural changes – health behaviours
Short-stay crisis units for mental health patients on crisis care pathways: systematic review and meta-analysis	2022	Anderson K et. al	Systematic review and meta-analysis	Mitigations – intervention
How can we decide a fair allocation of healthcare resources during a pandemic?	2021	Roadevin C and Hill H	Review	Context setting—healthcare
Socioeconomic inequalities in suicide mortality in European urban areas before and during the economic recession	2020	Borrell C et. al	Ecological study of trends	Short, mid and long term health implications – mental health
COVID-19 and Ethnic Inequalities in England and Wales	2020	Platt L and Warwick R	Analysis of mortality, case data and labour force data	Context setting – income shock
Suicide risk assessment in UK mental health services: a national mixed-methods study	2020	Graney J et.al	Mixed methods study	Short, mid and long term health implications and mitigations – mental health – intervention
Did the UK policy response to Covid-19 protect household incomes?	2021	Brewer M et. Al	Analysis of UK policy response to COVID-19	Context setting- economics
Pay (for it) as you go: Prepaid energy meters and the heat-or-eat dilemma	2022	Burlinson A et.al	Analysis of longitudinal survey	Context setting and Short, mid and long term health implications– economics and nutrition
Acute day units in non-residential settings for people in mental health crisis: the AD-CARE mixed-methods study	2021	Osborn D et.al	Mixed methods – survey, cohort study and qualitative interviews	Mitigations – mental health, intervention
After the storm, Solar comes out: A new service model for children and adolescent mental health	2021	Vusio F et.al	Case study of service model	Mitigations—mental health, children, intervention
Changes in care costs associated with cognitive behavioural therapy for psychosis delivered in routine clinical practice	2020	Sheaves B et.al	Analysis of inpatient care and out of hours support	Mitigations – CBT mental health
The National Health Service (NHS) in 'crisis': the role played by a shift from horizontal to vertical principles of equity	2020	Asthana S and Gibson A	Review and commentary	Context setting– NHS coastal populations
Precarious employment and workplace health outcomes in Britain	2023	Haile GA	Analysis of British Workplace Employment Relations Survey	Context setting – economics and population health
The psychiatric decision unit as an emerging model in mental health crisis care: a national survey in England	2021	Goldsmith LP at.al	National (England) survey	Mitigations – intervention, mental health
Tackling fuel poverty through facilitating energy tariff switching: a participatory action research study in vulnerable groups	2013	Lorenc, A	Participatory Action Research, using semi-structured interviews	Mitigations – elderly, housing, intervention
Austerity policy and child health in European countries: a systematic literature review	2020	Rajmil, Luis et. al	Systematic literature review	Behavioural changes – children, economics
The possible impact of increased physical intimate partner violence during the COVID-19 pandemic on ocular health	2021	Hicks, Patrice M. et.al	Review	Short, mid and long term health implications – domestic violence
Housing and Healthy Child Development: Known and Potential Impacts of Interventions	2020	Dunn, James R. et.al	Review	Mitigations– housing
Economic evaluations of videoconference and telephone consultations in primary care: A systematic review	2021	De Guzman et.al	Systematic review	Mitigations– intervention
Child poverty and health inequalities in the UK: a guide for paediatricians	2023	Lee, Alice R. et. al	Review	Mitigations -
Indirect health sector actions and supportive strategies to prevent malnutrition	2020	Keats, Emily C. at.al	Review and commentary	Mitigations – intervention
Social determinants of health screening in paediatric healthcare settings	2023	Nerlinger, Abby L. and Kopsombut	Review	Behavioural changes—screening

## Results

The results are presented by the five objectives outlined in the methodology. Table 2 summarises the 26 papers identified as part of our review process and Table 3 describes the grey literature used.

**Table 2:** Summary of grey literature used during evidence review

Title	Year	Authors	Nature of grey literature
**Poverty and the health and care system: The role of data and partnership in bringing change**	2022	The Kings Fund	Partnership with Centre for Progressive Policy which looks to explore datasets to inform understanding and action on poverty and healthcare -forging links and good practice
**UK Poverty 2023: The essential guide to understanding poverty in the UK**	2023	Joseph Rowntree Foundation	Recent trends in poverty in UK, with variation in regions and population
**Cost of living crisis**	2022	Institute for Government	Explanation and predictions of inflation and cost of living
**Cost of living crisis in Wales** **A public health lens**	2022	Public Health Wales	Describes the impact of cost of living and its impact on health and wellbeing
**Press Release Fuel Poverty, Cold Homes and Health Inequalities in the UK**	2022	Institute of Health Equity	Predictions of the impact of fuel poverty on the health of children
**Fuel poverty: updated estimates for the UK**	2022	Child and Poverty Action Group	Analysis to provide estimates of fuel poverty in the UK
**The cost of caring: poverty and deprivation among residential care workers in the UK**	2022	The Health Foundation	Poverty within the care worker population
**“Heart-breaking and wrong” that a million children under 4 growing up in poverty—JRF**	2023	Joseph Rowntree Foundation	Focus on impact of poverty of children and families
**Food Insecurity Tracking**	2020–2023	The Food Foundation	Impact of food insecurity, via survey, on households in the UK. Focus on groups such as families, ethnic groups, people with disabilities and those on benefits
**The Road Ahead 2023: The ongoing impact of cost of living**	2023	NCVO	Impact of cost of living on charities and voluntary sector
**Charity Sector Cost Of Living Crisis—Funding Cuts Report**	2023	Charity Excellence Framework	Impact of cost of living on charities and voluntary sector
**Cost of Living: Armed Forces Personnel**	2022	UK Parliament	Impact of cost of living on military personnel
**Rising cost of living in the UK**	June 2023	UK Parliament	Details rising process, including food and energy and available government support
**Impact of increased cost of living on adults across Great Britain: September 2022 to January 2023**	2023	ONS	Analysis of those affected by an increase in cost of living
**Give free school meals to all primary children, school nurses urge**	2023	Nursing Times	Impact of cost of living on school children
**Employment in the UK: July 2023**	2023	ONS	Details economic inactivity in UK
**Monthly mortality analysis, England and Wales**	2023	ONS	Monthly mortality statistics for UK
**Child food insecurity doubles fuelling calls for urgent expansion of Free School Meals**	2023	The Food Foundation	Policy commentary – focus on food insecurity for families with children
**Anxiety nation: Britain’s epidemic of mental health problems and rampant economic insecurity**	2022	Joseph Rowntree Foundation	Exploring connections between financial insecurity and poor mental health
**Record number of emergency food parcels provided to people facing hardship by Trussell Trust food banks in past 12 months**	2023	The Trussell Trust	Statistics for the use of Foodbanks in the UK
**Impact of increased cost of living on adults across Great Britain: February to May 2023**	2023	ONS	Analysis of those affected by an increase in cost of living

**Table 3:** NOS scoring of eligible studies

AUTHOR	SELECTION					COMPARABILITY	OUTCOME			NOS TOTAL (0–10)
Osborn et al.,	1	1	0	2	**4/5**	**2/2**	2	1	**3/3**	**9/10**
Goldsmith et al.,	1	0	0	1	**2/5**	**0/2**	2	0	**2/3**	**4/10**
Lorenc et al.,	1	0	0	0	**1/5**	**2/2**	0	2	**2/3**	**5/10**

### Current CoL crisis

We have addressed objective 1 of the evidence review (describe the current CoL, including the causes, triggers, and drivers of economic instability by internal and external shock factors) as part of the introduction of this report and cited findings from the World Health Organisation [2], the Bank of England [11] and the Institute for Health Equity [14]. Six (out of twenty-six) peer-reviewed papers from the evidence review were also used to gather evidence and establish a baseline understanding of the current CoL crisis.

### Short, mid and long term implications

The review mapped health and wellbeing outcomes using evidence published from six (out of twenty-six) peer-reviewed papers from the evidence review and findings from the World Health Organisation [2], the King’s Fund [3], the Joseph Rowntree Foundation [9, 20] and Public Health Wales [13]. A summary of the findings from our reading across papers and grey literature, as well as discussions with our expert advisory panel, on relatable health outcomes is shown in Table 4 and are themed by type of impact (environmental impacts, mental health, physical health and service pressures/impact areas), by time (immediate 1–2 months, intermediate 3–6 months and longer-term 6 months and over) and populations at risk.

**Table 4:** Summary of health related outcomes

	Immediate (1-2 months)	Intermediate (3-6 months)	Longer-term (6+ months)	Populations most at risk
Environmental	• Living in cold/mouldy homes • Material depravation [21] (e.g., inability to buy clothes or food) • Unsafe sleeping practices with infants and younger children	• Increased risk of hospital admissions from: Acute respiratory illness, Coronavirus/Flu/ Influenza-like illnesses • Increased A&E attendance from: Acute respiratory illness, Coronavirus/Flu/ Influenza-like illnesses • Increased prevalence of hypertension	• Increased morbidity and mortality rates from respiratory illness • Increased prevalence of COPD • Increased prevalence of cardiovascular disease	• Intergenerational living (urban) • Children (under 18 years) [22, 23] • Elderly (over 65 years of age) • Co-morbidities / disability
Mental health	• Anxiety, Depression, reduced quality of life metrics • Insomnia[24] • Self-harm behaviour/ Suicide [25] • Alcohol & Substance Misuse • Diminished social connectedness • Unhappiness/ Dissatisfaction with life	• Increased GP attendance • increased hospital admissions • Increased A&E attendance • Increased use of psychotropic drugs [26] • Increased workforce sickness / absence from work	• Increased mortality rates from suicide/self-harm behaviours • Increased hospital admissions from liver cirrhosis • Increased mortality rates from Alzheimer’s/Dementia	• Young people • Working age adults(in families father’s mental health affected more than mothers in economic shocks) [27] • Young males – suicide
Physical health	• Physical pain e.g. musculoskeletal pain, chest pain • Falls/Trips/Injuries • Coronavirus/Flu • Malnutrition, particularly in children • Increased risk of infections • Increased risk of domestic violence [28]	• High Blood Pressure • Transient Ischaemic events (TIA); myocardial infarctions (MI) • Decrease in healthy food consumption [29] e.g. increase in obesity [30] • Fainting due to hunger, stunted growth, increased anxiety in children [31] • Complications of malnutrition for example, vitamin deficiencies • Increased hospital admission from food poisoning • Increase in A&E admissions from falls/trips/injuries	• Increased mortality rates from respiratory illness, cardiovascular diseases, and infectious diseases • Excess mortality rates including stroke • Increased mortality rates from Alzheimer’s/Dementia	• Children • Elderly • Co-morbidities / disability
Service pressures / impacts	• NHS 111 calls (respiratory difficulties), flu symptoms • Increase in infectious diseases in A&E settings • Reduced uptake of screening and immunisation programmes • People not accessing services (rural) • Use of food banks • Increased homelessness	• Increased use of medications e.g. asthma medication in children, painkillers, or anxiety medications in adults, increase in antibiotic prescription • Increased A&E attendance from acute respiratory infections • Increased A&E admissions from stroke, TIA, and MI • Reduced uptake of screening and immunisation programmes [32]: for example; breast cancer, cervical cancer, aortic aneurysm • Increase in infectious diseases in A&E settings • Reduced workforce capacity due to illness	• Excess all-cause and cause specific mortality, for example, cardiovascular disease • Increased mortality rate from; breast cancer, cervical cancer, aortic aneurysm • Increase in children in care [33]	• Rural • Children • Elderly • Co-morbidities / disability • Working age – increased workforce sickness and absenteeism

The review found that the strongest evidence for the impact of a CoL crisis, particularly from living in cold homes, was on acute hospital admissions due to respiratory distress or illnesses and in particular, affected the very young (children aged 0–4 years) or the elderly (75 + years) [13, 16, 29, 33]. It is also anticipated that in 2023, a further 500,000 children across England will fall below the UK poverty line [33] with families struggling to buy essential items like food and clothing [34]. Increases in the cost of energy and food would result in families choosing between energy dense foods vs. more costly healthier food options [30]. The CoL crisis could widen socioeconomic inequalities in obesity by affecting disadvantaged families and communities at an existing risk of obesity [30]. Specifically concerning in younger children are issues related to living in a cold home – including unsafe sleep practices for children, reduced ventilation to keep ‘the heat in’, and living in areas where it is unsafe to open windows [35].

### Behaviour change

Objective 3 of the evidence review was on the impact of the CoL crisis on health behaviours, how people cope or respond to a CoL crisis, and how it impacts on their self-care e.g., attending regular health checks or screening visits. Evidence from 3 (out of 26) peer-reviewed papers [32, 36, 37] from the evidence review and findings from Public Health Wales [13] informed our results. These were:

- Reducing transport related costs associated with attending screening services and appointments may be effective as cost was a barrier for people accessing health and care services. Missing or delaying medical appointments will exacerbate physical and mental health illnesses and delay treatment.
- Economic crisis was associated with a lower probability of drinking alcohol frequently and lower probability of being physically inactive.
- Economic austerity was associated with increasing child poverty and poorer access and quality of services provided, particularly to children with physical disabilities.
- Screening could be offered in acute care and community-based settings to address the social needs of vulnerable patients and families.

The Food Foundation has tracked food insecurity [38] and found that:

- 17.7% of households experienced food insecurity (moderate or severe) in January 2023, with 24.4% of households with children experiencing food insecurity.
- 3.2 million adults (6.1% of households) reported not eating for a whole day because they couldn’t afford food.
- Key workers are more likely to be experiencing food insecurity.
- Half of households on Universal Credit experienced food insecurity
- Disability exacerbates food insecurity.
- Non – white people more likely to experience food insecurity.

### Mitigation strategies

Objective 4 of the evidence review was on potential interventions to address the CoL crisis at a population level. Nine (out of 26) peer-reviewed papers [39–47] were included alongside evidence from the grey literature sources including the King’s Fund [3], Joseph Rowntree Foundation [9] and Public Health Wales [13].

Where possible, scoring of studies with an intervention were undertaken. Three studies were scored using the Newcastle Ottawa Scale (NOS) as detailed in Table 1:

A summary of interventions that could be deployed to mitigate the effects of the CoL crisis is presented in Appendix 2 with most peer-reviewed publications focussing on mitigating the negative impacts on mental health. By theme and strength of evidence (where grading was possible), the key findings are:

#### Mental health

- Strong evidence (based on systematic review and meta-analysis of results) to support the use of short-stay crisis units for people experiencing mental health crises.
- Evidence showed that suicide risk assessment tools are deployed variably across 85 NHS mental health organisations with limited staff training. Study outcomes recommend standardisation of assessment tools and bespoke staff training in use and implementation, to help improve service and care provision for people attempting suicide or serious self-harm.
- Despite strong study design, there was a lack of evidence for the effectiveness of specialist mental health day units compared to crisis teams which support people in crisis at home.
- Similarly, there was weak evidence for the use of mental health units which act independently of emergency departments with some units experiencing lengthy stays in a setting which was designed for short stays.
- Public Health Wales 2023 [13] related commissioned review recommends focussing on mental health and wellbeing support including suicide prevention campaigns (short term) and linking people with community support including voluntary and community sector (long term).

#### Physical health

- Providing financial help and home improvements to those at risk of living in a cold home (increasing the warmth and/or energy efficiency of a home)
- Preventing falls through exercise (strength training) and home safety assessments
- Preventing the spread of respiratory viral infections maximising vaccination uptakes e.g., influenza vaccines, employers encouraging sick employees to stay at home, and providing handwashing advice.
- Helping vulnerable individuals keep warm, particularly those experiencing homelessness.

#### Environmental (income support, energy relief, housing, food)

- Public Health Wales recommend providing targeted support on energy bills and extending the Winter Fuel Support Scheme for all households and focusing on the elderly, the very young and people with disabilities or long-term health conditions.
- Advising on modifying home energy use in the community e.g., best time of day to use appliances or monitoring use with a smart meter.
- Extension of the council tax reduction scheme for tenants and households experiencing hardship
- Provision of meal allowances and free school meals to all primary school children.
- Supporting emergency schemes such as food banks and community groups which provide essentials of daily living (extend to community/faith groups).

#### Voluntary & community sector

- Weak evidence for the use of individualised/bespoke advice on facilitating energy tariff switching, particularly in vulnerable communities (BAME, elderly over 75 years, and families with young children). Young families most likely to switch, elderly least likely due to apathy, lack of knowledge and scepticism.

### Impact on system partners

The last objective of the evidence review aimed to summarise the impact of the CoL crisis on other partners in the health and care systems, such as the voluntary and community sector, fire and rescue service, military, police, and ambulance service. The results of the review (predominantly grey literature as well as discussions with our expert advisory panel) found the following impacts by different sectors as presented here:

#### Voluntary and community sector

- There has been a reduction in voluntary services and community groups, particularly in deprived areas where the level of need is higher. This is largely driven by an increase in energy prices, consumable such as food, and an increase in fuel prices, and more expensive labour [48].
- Reductions in charitable donations and volunteer time have been seen in charity settings with charities now responding more to crisis planning, including welfare and wellbeing support for people.
- A decrease in the volume of food donated to food banks has resulted in limited supplies of food provisions against rising demand in the community [49].
- The rate of closures and number of closures of charities was significantly higher in 2022 than in 2021, and simultaneous reduction in the resilience of the voluntary and charity sector in April 2023 compared with previous years [50]. This includes operating losses for large front-line charities.
- There has been a reduction in number of volunteers as part time jobs [50].
- Successes were seen, with services being continued, in charities providing hot meals to elderly residents and a scheme for free school meals provision during school holidays.
- Providing safe, warm spaces by making use of local amenities have been successfully deployed in some areas (Wiltshire Community Foundation).

#### Police

- Increases in fuel theft and shoplifting (crime/policing) were noted, however, the Office for National Statistics reports that this increase could be due to improved recording processes and practices by police staff and expansion of recorded crime figures to include new offences [51].

#### Fire & rescue

- Increased risk of fires as people try to heat their homes or find alternative and cheaper ways to light or heat their homes, for example, wood burner fires linked to chimneys not being swept [52].
- Increased emergency admissions in A & E from burns as a result of alternative heating mechanisms used or unsafe practices, for example, plugging in an electric heater too close to flammable materials.

#### Military

- Data from the House of Commons [53] shows that a series of measures have been introduced to mitigate against the CoL increase for defence people, veterans and service families including subsidised accommodation charges at 1%, freezing food charges, increasing travel allowance, and providing additional wraparound childcare services.

## Discussion

This is one of the first narrative reviews of the published and grey literature from 2020–2023 to describe the breadth of impact of the current and unique CoL crisis on population health in the UK. The main findings of this report refer to the immediate impacts on population health and well-being, including physical, mental, and financial health. This paper sequentially assesses the literature to present mapped population impacts, individual and population behavioural responses to the cost of living crisis, and the system wide implications of these impacts. Since conducting our review Broadbent et al. [54] and Richardson et al. [55], have published work which also investigates the impact of the cost-of-living crisis on population health, which corroborate our findings. Our review was produced to help stakeholders and partners in the health and care sector to rapidly assimilate key information, knowledge, and evidence on the impacts of the cost of living and use it to inform, challenge, change, and drive local policies and practices on tackling this crisis.

### The unique provenance of the cost of living

The COVID-19 pandemic, Brexit, and the Ukraine-Russia war have resulted in unique economic shocks in the UK with steep declines in GDP, reduction in disposable incomes, and increases in inflation from 2020 to 2023. The situation has been further compounded by public sector strikes, political instability, changing fiscal and energy policies, climbing interest rates, findings further corroborated by Broadbent and colleagues [54]. At a population level, the evidence reflects a reduction in spending power due to the rising costs of essentials such as food and medication, and basic utilities such as heating, electricity, and council tax [56]. Evidence from the grey literature, the Office of National Statistics, reports that from 17 to 29 May 2023, 7 in 10 adults reported an increase in their cost of living compared to the previous year, largely driven by food bills (95%), gas or electricity bills (73%) and fuel prices (39%) [31]. A reduction in a household’s spending power or income, is likely to have marked effects on health, as other longitudinal analyses and reports have purported [55].Our review highlights the unique provenance of the current cost of living crisis, and why it is markedly different and more serious compared to previous economic crises, both at a UK and global level.

### The short, mid- and long-term impacts

Our review captures a breadth of physical and mental health conditions which can be affected by the CoL crisis in the short, mid and long term. In the short term, the strongest evidence was for the impact of CoL on housing, resulting in cold, damp, or mouldy homes, and the subsequent effect on the rate of respiratory conditions. These impacts are also more likely to affect those already vulnerable, such as those with chronic conditions, lone parents, multigeneration families, and children [13, 16, 29, 33, 57]. Another group vulnerable to the CoL impacts include key workers, those on universal credit, disabled and people from non-white ethnicities [16, 21, 31]. The July 2023 labour market figures form the ONS show that over 410,000 people were not actively seeking employment due to long-term sick leave [58]. In the longer term, increased morbidity rates and mortality rates from all-causes and cause-specific, such as respiratory and cardiovascular diseases, are expected with recent publications citing an increase in premature mortality by up to 6.4% and life expectancy to decrease by 0.9% [55].This has been demonstrated with official mortality rates in March 2023, which were 4.8% above the expected rate [59].

Behaviour changes because of the CoL crisis are likely to confound the issues that are affecting physical and mental health. Changes in food buying patterns, changes in the frequency and temperature to which homes are heated, and reduced physical activity levels can contribute to poor physical and mental health and impact wellbeing. Other impacts include the inability to afford travels costs to attend screening or hospital services or the reduction in community services such as community pharmacies [13]. This CoL crisis has had a negative impact on voluntary services and community groups. Charity and food donations have decreased, whilst the need for charity and food banks have exponentially increased. The Trussell Trust report that from March 2022-March 2023, they provided almost 3 million emergency food parcels, higher than during the pandemic and more than double the number in the same period 5 years prior [60], a finding further highlighted by Broadbent and colleagues [54].

### What can be done to mitigate the impacts?

Our evidence suggests that financial help should be provided to those most at risk of living in a cold home because of the CoL crisis, such as lone parents, multigenerational families, and those living alone via targeted support for energy bills with a focus on those at risk from the CoL crisis [13]. In addition to these groups, Broadbent [54]and Richardson [55] also suggest focus on women, unemployed people and those who are living with a disability, as being most vulnerable to the cost of living crisis. Financial support could also be provided in terms of reduction of council tax, provision of universal free school meals [61], and bespoke advice on how to save energy when using appliances at home [13]. To mitigate against the effects of the CoL crisis on physical health, particularly from respiratory illness, evidence was suggestive of investing in-home improvements for those living in cold homes, strength training to reduce the risk of falls, and that flu vaccination uptake should be maximised to reduce the spread of influenza alongside good hand washing guidance [13]. Campaigns aimed at suicide prevention and linking people with community support may also benefit those with mental health illnesses and those who live alone. Our evidence also found that linking system partners across the health and social care arena and including community-based partners such as the voluntary, charity and faith sectors may collectively have better outreach and impact.

### What are the strengths and limitations of this review?

The key strength of this review is the immediate availability of a succinct report which considers the volume of evidence, both published and grey, on the current cost of living crisis. The authors also expanded the original search to include mitigating actions, behaviours, and strategies, so that colleagues may benefit from evidence-based solutions that may work at a population level. We have attempted to synthesis the most recent evidence, to build a picture of why the current economic crisis is unique, and what that means for the population. As with all original reviews, there are several limitations to this work. Firstly, the review period covers 2020–2023, rather than considering the impacts of historic CoL crises on population health. Whilst more evidence on population health outcomes may be needed, the provenance of the current CoL crisis remains unique and warranted specific focus and attention, hence our selection of the time window for review. Secondly, we were not able to ascertain peer-reviewed literature on the impacts on system partners including Fire & Rescue colleagues as these data remain unpublished, anecdotal, and discussed in privileged meetings. However, as more evidence gets published through this crisis, we may consider updating our review in due course to reflect the published evidence. Another limitation is the lack of consideration for any positive impacts of a cost of living crisis, for example a reduced consumption of alcohol due to reduced affordability, reduced usage of public transport due to costs, and a reduction in air quality in major cities. Lastly, due to the time, resources and need for this review, it was conducted in a 3-month timeframe to be of immediate use, value, and impact for current system partners but may have introduced bias to our findings such as a publication bias due to the shorter timeframe [62]. However, to mitigate this, we have provided a detailed description of methods used including search strategies and discussed the implications of the chosen method in terms of bias. Should more time and resources be made available, future iterations of this review should consider the wider impacts of the CoL crisis at a personalised and individual level, and family and community level but also include different types of study designs rather than the focus on reviews alone in this rapid synthesis which may not fully capture data on the cost of living impacts.

## Conclusion

At a population level, the current CoL affects everyone, but particularly exacerbates poorer physical and mental health outcomes in those already vulnerable in our society. Our review found that while the most vulnerable are people living alone, single parents and those living in multigenerational households, more can be done at a community and societal level to support and improve health outcomes. This review brings together the evidence to enable decision makers to act at the right time whilst targeting their resources at the right groups.

### Supplementary Information

**Additional file 1:** **Appendix 1.** Search Strategy. **Appendix 2.** Interventions to mitigating the impacts of the CoL crisis.

## Data Availability

All data used or analysed during this study are included in this published article and its supplementary information files.
