# Supplementary material for: The impact of the cost-of-living crisis on population health in the UK: rapid evidence review
Source: BMC Public Health. 2024 Feb 22;24:561. doi: 10.1186/s12889-024-17940-0 (PMC10882727; doi:10.1186/s12889-024-17940-0)
Supplement: Supplementary file 1 — Additional file 1: Appendix 1. Search Strategy. Appendix 2. Interventions to mitigating the impacts of the CoL crisis. [file 12889_2024_17940_MOESM1_ESM.docx]

Appendix 1: Search Strategy

**Embase - 24/02/2023**

1.           exp *poverty/

2.           poverty.ti,kw. or poverty.ab. /freq=3

3.           cost* of living.tw,kw.

4.           *food insecurity/

5.           food insecurity.tw,kw.

6.           food security.tw,kw.

7.           food poverty.tw,kw.

8.           energy poverty.tw,kw.

9.           fuel poverty.tw,kw.

10.         cold home*.tw,kw.

11.         (heating adj3 home*).tw,kw.

12.         *income/

13.         exp *household income/

14.         income security/

15.         (income adj7 change*).tw.

16.         (income adj3 (fall* or drop* or decreas* or los*)).tw.

17.         "cost of living"/

18.         living cost*.tw,kw.

19.         (household* cost* adj3 (increas* or ris*)).tw.

20.         financial* insecur*.tw,kw.

21.         economic stress*.tw,kw.

22.         economic* insecur*.tw,kw.

23.         ((increas* or rise or rising or rises) and ((cost* or price*) adj3 (energy or food or electric* or gas or heating or fuel or utilities or household*))).tw.

24.         (inflation adj5 (price* or cost* or energy or food or electric* or gas or heating or fuel or utilities or household)).tw.

25.         or/1-24

26.         exp *health/

27.         (health adj3 impact*).tw,kw.

28.         health.ti.

29.         ((physical or mental) adj health).ti,kw.

30.         well?being.ti,kw.

31.         exp *mental health/

32.         *hospitalization/

33.         *hospital admission/

34.         exp *health care utilization/

35.         exp *health care need/

36.         (hospital adj3 admission*).ti,kw.

37.         (hospitalised or hospitalized).ti,kw.

38.         (hospitalisation* or hospitalization*).ti,kw.

39.         (health service* adj3 (impact* or demand* or need* or "use" or usage or utili*)).tw.

40.         exp *chronic disease/

41.         *non communicable disease/

42.         long term condition*.ti,kw.

43.         (chronic* adj1 (disease* or ill* or condition*)).ti,kw.

44.         (fit* adj3 (employ* or work)).tw.

45.         economically active.tw,kw.

46.         ability to work.tw,kw.

47.         able to work.tw,kw.

48.         (return* adj3 work*).tw,kw.

49.         return* to productivity.tw.

50.         *return to work/

51.         *work resumption/

52.         *work capacity/

53.         *employment status/

54.         *unemployment/

55.         mortality.ti,kw.

56.         exp *mortality/

57.         or/26-56

58.         25 and 57

59.         limit 58 to (english language and yr="2000 -Current")

60.         limit 59 to "reviews (maximizes specificity)"

**HMIC - 24/02/2023**

1.           exp poverty/

2.           poverty.ti. or poverty.ab. /freq=3

3.           cost* of living.tw.

4.           food insecurity.tw.

5.           food security.tw.

6.           food poverty.tw.

7.           energy poverty.tw.

8.           fuel poverty.tw.

9.           cold home*.tw.

10.         (heating adj3 home*).tw.

11.         income/

12.         low income/

13.         (income adj7 change*).tw.

14.         (income adj3 (fall* or drop* or decreas* or los*)).tw.

15.         exp "cost of living"/

16.         living cost*.tw.

17.         (household* cost* adj3 (increas* or ris*)).tw.

18.         financial* insecur*.tw.

19.         economic stress*.tw.

20.         economic* insecur*.tw.

21.         ((increas* or rise or rising or rises) and ((cost* or price*) adj3 (energy or food or electric* or gas or heating or fuel or utilities or household*))).tw.

22.         (inflation adj5 (price* or cost* or energy or food or electric* or gas or heating or fuel or utilities or household)).tw.

23.         or/1-21

24.         exp Health/

25.         (health adj3 impact*).tw.

26.         ((physical or mental) adj health).tw.

27.         well?being.tw.

28.         mental health/

29.         exp hospitalisation/

30.         health demands/

31.         health service provision/

32.         service demand/

33.         (hospital adj3 admission*).tw.

34.         (hospitalised or hospitalized).tw.

35.         (hospitalisation* or hospitalization*).tw.

36.         (health service* adj3 (impact* or demand* or need* or "use" or usage or utili*)).ti.

37.         chronic disease/

38.         non communicable diseases/

39.         long term condition*.tw.

40.         (chronic* adj1 (disease* or ill* or condition*)).ti. or (chronic* adj1 (disease* or ill* or condition*)).ab. /freq=3

41.         (fit* adj3 (employ* or work)).tw.

42.         economically active.tw.

43.         ability to work.tw.

44.         able to work.tw.

45.         (return* adj3 work*).tw.

46.         return* to productivity.tw.

47.         re employment/

48.         employment/

49.         exp unemployment/

50.         mortality.ti.

51.         exp mortality/

52.         or/24-51

53.         23 and 52

54.         limit 53 to yr="2000 -Current"

55.         limit 54 to article

**Medline - 24/02/2023**

1.           exp *Poverty/

2.           poverty.ti,kw. or poverty.ab. /freq=3

3.           cost* of living.tw,kw.

4.           exp Food Insecurity/

5.           food insecurity.tw,kw.

6.           food security.tw,kw.

7.           food poverty.tw,kw.

8.           energy poverty.tw,kw.

9.           fuel poverty.tw,kw.

10.         cold home*.tw,kw.

11.         (heating adj3 home*).tw,kw.

12.         *Income/

13.         (income adj7 change*).tw.

14.         (income adj3 (fall* or drop* or decreas* or los*)).tw.

15.         living cost*.tw,kw.

16.         (household* cost* adj3 (increas* or ris*)).tw.

17.         financial* insecur*.tw,kw.

18.         economic stress*.tw,kw.

19.         economic* insecur*.tw,kw.

20.         ((increas* or rise or rising or rises) and ((cost* or price*) adj3 (energy or food or electric* or gas or heating or fuel or utilities or household*))).tw.

21.         (inflation adj5 (price* or cost* or energy or food or electric* or gas or heating or fuel or utilities or household)).tw.

22.         or/1-21

23.         exp *Health/

24.         (health adj3 impact*).tw,kw.

25.         health.ti.

26.         ((mental or physical) adj health).ti,kw.

27.         well?being.ti,kw.

28.         *Mental Health/

29.         *Hospitalization/

30.         *Patient Admission/

31.         *"Health Services Needs and Demand"/

32.         (hospital adj3 admission*).ti,kw.

33.         (hospitalised or hospitalized).ti,kw.

34.         (hospitalisation* or hospitalization*).ti,kw.

35.         (health service* adj3 (impact* or demand* or need* or "use" or usage or utili*)).tw.

36.         *Chronic Disease/

37.         *Noncommunicable Diseases/

38.         long term condition*.ti,kw.

39.         (chronic* adj1 (disease* or ill* or condition*)).ti,kw.

40.         (fit* adj3 (employ* or work)).tw.

41.         economically active.tw,kw.

42.         ability to work.tw,kw.

43.         able to work.tw,kw.

44.         (return* adj3 work*).tw,kw.

45.         return* to productivity.tw.

46.         *"Return to Work"/

47.         *Unemployment/

48.         mortality.ti,kw.

49.         exp *Mortality/

50.         or/23-49

51.         22 and 50

52.         limit 51 to (english language and yr="2000 -Current")

53.         limit 52 to "reviews (maximizes specificity)"

Appendix 2:Interventions to mitigating the impacts of the CoL crisis

| **Intervention Details** | **Authors, Publication, Date** | **Intervention Population** | **Administered by; Frequency of Intervention** | **Outcomes/ Impacts / Clinical Efficacy / Effectiveness** | **Limitations/ Caveats** | **Strength of Evidence (weak, moderate or strong) with NOS score if appropriate** | **Cost Effectiveness** |
| --- | --- | --- | --- | --- | --- | --- | --- |
| The use of **short-stay crisis units for people experiencing mental health** crisis, as opposed to short in-patient stays.  These units were defined as any mental health service that was hospital based; allowed overnight stays; length of stays were less than 1 week; and their objective was to stabilise and/or assess patients, with the aim of onward referral to reduce ED wait times and/or psychiatric admissions. | Anderson *et al*., 2022 | Adults | Maximum length of stay for units ranged from 24-hr to 96hr.  Staffing varied and were composed of multidisciplinary teams. These included emergency mental health clinicians; drug and alcohol clinicians; psychiatrists; psychiatry registrars; psychologists; nurses and mental health nurses; clinical staff trained in trauma-informed care. | Systematic review involving 12 studies, which reported on benefits across different outcomes.  Meta-analysis showed **significant reduction in length of stay in ED and ED wait times, and reduction in the number of in-patient admissions.**  As well, two studies reported decreases in the need for security services and restraint procedures; one study reported a significant decrease in the total time spent in care when in the crisis unit; one study reported a significant reduction in psychiatric hold rates. | Studies were at risk of bias; one study was identified at being at critical risk of bias and was excluded from meta-analysis.  Unable to undertake meta-analysis for every outcome due to differences in the way outcomes were reported between studies.  Unable to fully explore the impact of short-stay crisis units on patient experience and suicidality.  Limited evidence to consider the cost benefit. | Hierarchy of evidence (strong) as systematic review and meta-analysis | Limited evidence to consider the cost benefit. |
| 85 suicide risk assessment tools across 85 NHS mental health organisations - one from each organisation – were used in the analysis.  Across 85 NHS mental health organisations, there is little consistency among the use of risk assessment tools.  39% of Trusts used their own risk assessment tools; all Local Health Boards in Wales used the Wales Applied Risk Research Network Tool; all Trusts in Northern Ireland used the same risk assessment method.  Some Trusts used multiple tools (only the main tool used was used in the analysis). | Graney *et al.,* 2020 | Suicide Risk Assessment in Adults | Nurses. | Clinicians reported the tools were useful for documenting patient information and as a framework for discussion but felt training to use the tools was inadequate.  Only 33% of patients were aware risk assessment tools were being used. 52% of patients felt listened to, but 44% were felt “disregarded” and that the assessment was “impersonal”. 22% of patients felt the approach was inconsistent.  Only 47% of tools specifically collected data on suicidality, with one patient commenting the question is “passed over too quickly”  A common theme among carers was frustration toward a lack of involvement in crisis planning, with lack of clarity on how to manage situations at home. | Survey; risk of response bias. Respondents recruited through convenience sampling has risk of bias.  Low numbers of patient and carer respondents compared to clinicians.  Did not request verifying information.  Study advertised online, limiting catchment pool. | n/a | No data |
| The objective was to assess how effective Acute Day Units (ADUs) were in supporting mental health recovery compared to care provided by NHS Crisis Resolution Teams (CRTs). | Osborn *et al.,* 2021 | Adults | Administered by ADU and CRT staff.  Client Satisfaction Questionnaire, Short Warwick–Edinburgh Mental Wellbeing Scale, and Center for Epidemiologic Studies Depression Scale taken at 8- to 12-week time points. | The difference in rate of admission between ADU and CRT participants was not statistically significant.  However, Client Satisfaction Questionnaire scores showed higher satisfaction in those patients treated as part of the ADU.  ADU participants also had higher Short Warwick–Edinburgh Mental Wellbeing Scale scores (higher shows better wellbeing), and lower Center for Epidemiologic Studies Depression Scale scores (less is less depressed). | Possible recruitment bias.  Participants were not randomised.  Reliant on the quality of Electronic Health Records.  Could not include the patients most severely affected by mental health; findings cannot be generalised to this group.  Lack of previous ADU costing. | Strong evidence (9/10) | The difference in cost between the ADU and CRT groups throughout the duration of the study was negligible. |
| The 0-19’s “Solar” service is an “emotional and wellbeing mental health service”.  Uses an integrated, whole-system approach toward assessing and treating young people. The partners included: CAMHS, Barnardo’s primary mental health service ( crisis-home treatment team) and Autism West Midlands (learning disability support and education for service users).  A further aim is to bridge the transition from CAMHS to AMHS. | Vusio *et al.,* 2020 | Children and Young People | Multi-organisational; CAMHS, Barnardo’s primary mental health service ( crisis-home treatment team) and Autism West Midlands (learning disability support and education for service users). | No results – is a proposed model. |  | n/a | No data |
| Six Psychiatric Decision Units (PDUs) (also known as Psychiatric Emergency Services or Crisis Stabilisation Units) are open 24hrs a day, accept voluntary admissions only, provide recliners rather than beds, and limits stays to 12-72 hours.  They act as a service, independent of the ED and wards, where patients in mental health crisis can be assessed and treatment plans developed. | Goldsmith *et al.,* 2021 |  | Mental health nurses, senior nurses, and healthcare assistants.  High staff-patient ratio of 1:2. | Purpose was to deduce the prevalence and scope of PDUs.  NHS Trusts with a PDU were twice as likely to have several other crisis services and an acute day unite.  This may indicate that trusts which prioritise crisis care invest in PDUs.  However, several PDUs have been decommissioned due to poor quality reports. | Cross-sectional; provides information of PDUs at the time of completion but no trends/changes over time. | Weak evidence (4/10) | No data. |
| Facilitating energy tariff switching by providing personalised advice per household/family | Lorenc et al., | BME communities, older people (75+ years) and families with young children | Community researcher from voluntary and community organisations | Younger families most likely to switch, older people least likely. Key reasons including apathy, lack of time, fear or scepticism or loyalty. | Select sample population. Not representative of the general UK population. | Moderate evidence (5/10) | n/a |
